# Machine Learning Predicting Optimal Preparation of Silica-Coated Gold Nanorods for Photothermal Tumor Ablation

**DOI:** 10.3390/nano13061024

**Published:** 2023-03-12

**Authors:** Jintao Zhang, Jinchang Yin, Ruiran Lai, Yue Wang, Baorui Mao, Haonan Wu, Li Tian, Yuanzhi Shao

**Affiliations:** 1 State Key Laboratory of Optoelectronic Materials and Technologies, School of Physics, Sun Yat-sen University, Guangzhou 510275, China; 2 Institute of Biological and Medical Engineering, Guangdong Academy of Sciences, Guangzhou 510316, China; 3 State Key Laboratory of Oncology in South China, Collaborative Innovation Center for Cancer Medicine, Sun Yat-sen University Cancer Center, Guangzhou 510060, China; 4 Guangdong Provincial Key Laboratory of Magnetoelectric Physics and Devices, Sun Yat-sen University, Guangzhou 510275, China

**Keywords:** silica-coated gold nanorods, machine learning, process optimization, high-throughput prediction, photothermal tumor ablation

## Abstract

Gold nanorods (GNRs) coated with silica shells are excellent photothermal agents with high surface functionality and biocompatibility. Understanding the correlation of the coating process with both structure and property of silica-coated GNRs is crucial to their optimizing preparation and performance, as well as tailoring potential applications. Herein, we report a machine learning (ML) prediction of coating silica on GNR with various preparation parameters. A total of 306 sets of silica-coated GNRs altogether were prepared via a sol–gel method, and their structures were characterized to extract a dataset available for eight ML algorithms. Among these algorithms, the eXtreme gradient boosting (XGboost) classification model affords the highest prediction accuracy of over 91%. The derived feature importance scores and relevant decision trees are employed to address the optimal process to prepare well-structured silica-coated GNRs. The high-throughput predictions have been adopted to identify optimal process parameters for the successful preparation of dumbbell-structured silica-coated GNRs, which possess a superior performance to a conventional cylindrical core–shell counterpart. The dumbbell silica-coated GNRs demonstrate an efficient enhanced photothermal performance in vivo and in vitro, validated by both experiments and time domain finite difference calculations. This study epitomizes the potential of ML algorithms combined with experiments in predicting, optimizing, and accelerating the preparation of core–shell inorganic materials and can be extended to other nanomaterial research.

## 1. Introduction

Gold nanorods (GNRs) possess unique optical properties, such as tunable surface plasmon resonance and intense photothermal effects [1,2,3,4], enabling GNRs to be promising inorganic materials in biomedical applications including bioimaging [5,6,7], photothermal therapy [8,9,10,11], and drug delivery [12,13,14]. To improve the stability and biocompatibility of GNRs without affecting their surface reaction, researchers coated GNRs with silica for their high light transmittance [15]. Thanks to the refractive index sensitivity of GNRs, the surface plasmon resonance peak position and intensity of GNRs can be modulated by adjusting the silica shells [16]. Lim et al. found that a 20 nm silica shell shows the highest photothermal effect [17]. The morphology of silica shell impacts the performance of silica-coated GNRs significantly. The preparation of well-structured silica-coated GNRs is dependent upon numerous process factors in an experimental protocol, and these process factors pose an intricate challenge to a stable optimizing synthesis of well-structured silica-coated GNRs. Conventional investigation to prepare silica-coated GNRs can partially delineate the influence among various processes. For instance, the study of three key factors, cetyltrimethylammonium bromide (CTAB), GNRs, and tetraethyl orthosilicate (TEOS), can reveal the concept of the existence of silicon precursor-dependent regions and CTAB-dependent regions in the preparation of silica-coated GNRs. [18] However, the morphology of the silica shell can also be controlled by adjusting pH, silica source type, reagent concentration, coating time, reaction temperature, and other factors related to the hydrolysis and condensation rate of the silica source [19]. Conventional experimental methods to study all these factors are too costly and time-consuming. Thus, it is quite desirable to harness a low-cost and high-efficient method that can analyze all experimental variables.

Recently, machine learning (ML) has emerged as a powerful tool of materials development for analyzing massive amounts of data, predicting physical and chemical properties of materials effectively, and establishing a constructive process–structure–property relationship [20,21,22,23]. Atwood et al. reported that XGBoost is a powerful ML model for predicting the crystallization propensity of metal organic nanocapsules. A new structure of nanocapsule was successfully synthesized with the help of chemical feature scores and feature importance derived from the XGBoost classifier [24]. Raccuglia et al. reported the application of a support vector machine (SVM) algorithm to use the chemical space created by past successful and unsuccessful experiments to reveal new hypotheses regarding the conditions for successful product formation [25]. To the best of our knowledge, the preparation of silica-coated GNRs guided by the ML algorithm has not yet been reported. Moreover, the chemical insight gained from these reported ML models is still quite scant, e.g., the optimal process parameters relevant to the reaction. It is worth introducing ML into silica-coated GNR preparation to explore the process parameter design and predict the optimal process and property of silica-coated GNRs.

This study establishes a mapping relationship from process parameters to preparation outcomes to predict the optimal preparation of silica-coated GNRs. The XGBoost model is evaluated along with seven other models, and it arguably outperforms other models to attain the best prediction performance in all evaluation metrics. The XGBoost model also offers an insight into the preparation process according to the derived feature importance scores and decision trees and achieves a high throughput prediction on the basis of the optimized process features for a randomly generated virtual experimental group. Through statistical analysis of all successful groups among them, this study can obtain the best reaction conditions for each process parameter. Furthermore, a unique form of dumbbell silica-coated GNRs has been successfully prepared on the basis of those parameters suggested by the ML model. Both in vitro and in vivo photothermal experiments and time domain finite difference calculations show that the dumbbell silica-coated GNRs present a more excellent optical property than that of a conventional cylindrical core–shell counterpart, indicating the great potential of this dumbbell silica-coated GNR as an integrated platform for tumor therapy. The method reported in this study can also be extended to design and develop other composite nanostructure materials.

## 2. Materials and Methods

### 2.1. Materials

Gold(III) chloride trihydrate (HAuCl_4_·3H_2_O, ≥49% Au basis), cetyltrimethylammonium bromide (CTAB, ≥99%), sodium borohydride (NaBH_4_, ≥98%), silver nitrate (AgNO_3_, ≥99.0%), ascorbic acid (AA, ≥99.0%) and sodium hydroxide (NaOH, ≥98%) were purchased from Sigma Aldrich. Tetraethyl orthosilicate (TEOS, ≥99.0%) and Methanol (≥99%) was products of Macklin. Hydrochloric acid (HCl, 35–37%) were purchased from Acros Organics. Ethanol (≥99.7%) was purchased from Ghtech. Phosphate-Buffered Saline (PBS) were procured from Gibco, Switzerland. Propidium Iodide (PI) and Calcein-AM were purchased from Becton Dickinson Pharmingen, USA. Deionized water used throughout all experiments was ultra-pure Milli-Q water with an electrical resistivity of 18.25 MΩ·cm (25 °C). Carbon support films (300 eyes) for TEM purchased from Beijing XXBR Technology Co., Ltd.

### 2.2. Preparation and Characterization

GNRs were synthesized with the seed-mediated growth method [26,27]. Firstly, the seed solution was prepared by dissolving 0.364 g of CTAB into 9.17 mL of deionized water and adding 0.25 mL of HAuCl_4_ (0.012 mM). Then, 0.58 mL of ice-cold NaBH_4_ (20 mM) was quickly added into gold (III)-CTAB solution under vigorous stirring. After 2 min of stirring, the solution was maintained at 27 °C for 2 h. In the second step, the growth solution was prepared by dissolving 1.82 g of CTAB into 44 mL of deionized water with mild stirring, followed by adding 3 mL of HAuCl_4_ (0.012 mM), 1.04 mL of AgNO_3_ (5 mM), 0.4 mL of HCl (37%), and 1.64 mL of ascorbic acid (50 mM) into the stirred solution. Subsequently, the yellow solution became colorless, and 50 μL of the prepared seed solution was added to the growth solution with 10 s of gentle stirring. Finally, the solution was kept undisturbed at 27 °C for GNR growth for 12 h. The resultant mixture was washed by centrifugation, followed by removing the supernatant to remove the remaining reactants.

The silica coating process was performed using the previously-reported Stöber method with slight modification [28,29]. An amount of 10 mL of GNRs colloidal solution was washed and centrifuged twice and then dispersed into 15 mL of a specific concentration of CTAB solution. Then, 0.1 mL of NaOH solution (0.1 M) was added to the GNR solution. After gentle stirring for 20 min, 300 μL of TEOS alcohol solution (25 vol%) was added three times with 30 min intervals under mild stirring. The mixture was aged for 12 h with stirring and then the prepared silica-coated GNRs were centrifuged and washed with deionized water two times.

Transmission electronic microscope (TEM) samples were prepared by dripping the silica-coated GNR solution onto carbon-coated copper grids and drying the solvent. TEM images and energy dispersive X-ray detector (EDX) spectra were recorded using an FEI Tecnai G2 Spirit instrument (FEI, The Netherlands) operating at 120 kV. High-resolution TEM (HR-TEM), selected area electron diffraction (SAED), high-angle annular dark-field scanning TEM (HAADF-STEM), EDX elemental mapping, and line scanning were recorded on a Tecnai G2 F30 transmission electron microscope (FEI, The Netherlands) at an accelerating voltage of 300 kV. An ultraviolet–visible–near infrared (UV–Vis–NIR) spectrophotometer (UV-3600, SHIMADZU, Japan) was employed to collect the sample’s absorption spectra at wavelengths from 400 nm to 1200 nm.

### 2.3. Experimental Parameter Preprocessing and Feature Engineering

Feature engineering aims to transform raw data into more suitable and representational forms for ML algorithms. According to the TEM images of silica-coated GNRs, they were subsumed into two categories: successful coating and unsuccessful coating. Finally, 306 sets of data were collected and collated for ML. The input features of the constructed dataset are listed in Table 1, including continuous and categorical variables. For the continuous variables, a data normalization procedure was performed to ensure the equal contribution of each parameter based on the MinMax algorithm:

(1) $$\;X_{norm}=\frac{X-X_{min}}{X_{max}-X_{min}}$$

where $\;X_{norm}$, X, $X_{max}$, and $X_{min}$ are the normalized values, the true value of the variable, and the maximum and minimum values of the dataset, respectively.

Categorical variables include ordinal categorical variables and non-ordinal categorical variables. Ordinal categorical variables are a very common variable form of variable that usually has multiple possible values with hierarchical relationships between the values. For example, the stirring intensity during the aging process is coded as 0 without stirring, 1 and 2 with a slow and fast stirring, respectively. The non-ordinal categorical variable is mainly related to the solvents of TEOS, which we adopted to denote the solvents for TEOS, ethanol, and isopropanol in this study. For the convenience of ML, they need to be numerically processed. Target encoding was performed for non-ordinal categorical variables, which is a coding method that derives numerical replacement category features from the target [30].

### 2.4. Machine Learning Models and Evaluation Metrics

Eight different ML algorithms were employed to train models for predicting the preparation of silica-coated GNRs, i.e., logistic regression (LR), ref. [31] support vector machine (SVM) [32], k-nearest neighbors (KNN) [32], decision tree (DT) [33], adaptive boosting (ADA) [34], random forest (RF) [35], gradient boosting decision tree (GBDT) [36], and eXtreme gradient boosting (XGBoost) [37]. All of those ML models were created using python with the scikit-learn package and the xgboost package. In the hyperparameter tuning process of the model, we performed a continuous grid search for a subset of hyperparameters and employed 5-fold cross-validation to prevent overfitting. The search space of the hyperparameters adjusted for each model and the resulting optimal parameters are listed in Table S1.

To evaluate the model’s generalization ability, we introduced five evaluation metrics, accuracy (ACC), receiver operating characteristic (ROC) curve, the area under ROC curve (AUC), precision, recall, and F1-score. The properties of the successful coating were classified as positive, whereas the properties of the unsuccessful coating were classified as negative. AUC value can be used to intuitively assess the discriminatory ability of a classifier. An AUC value of 1.0 indicates perfect discrimination between positive and negative samples, while an AUC value of 0.5 indicates no discriminatory ability. The recall was calculated by dividing the number of predicted true positive samples against the total number of actual positive samples. The precision indicates how many of the samples predicted to be positive are truly positive, while the recall indicates how many of the positive cases in the sample were predicted correctly. F1-score is considered a kind of reconciled average of the model accuracy and recall. The best model is usually the result of a thorough examination of each evaluation metric. All data were relabeled using the prediction results of the XGBoost model and the prediction results were modeled with decision trees. An easy-to-analyze decision tree was generated by this method [38].

### 2.5. Time Domain Finite Difference Simulation

The whole GNR (16 × 53.5 nm^2^) with the refractive index provided by Johnson and Christy was embedded in a 42 nm thick layer of silica with a refractive index of 1.5 to create a cylindrical model. The corresponding dumbbell model was constructed with the same GNR and its two ends were embedded inside a 42 nm thick silica sphere. The models were placed in a background solution with a refractive index of 1.33. The total field scattered field source was set as incident along the x-axis at a wavelength of 800 nm and polarized along the longitudinal direction of the GNR. The grid step was set to 1 nm. The electric field distribution and charge of the models were calculated numerically.

### 2.6. In Vivo and In Vitro Photothermal Experiments

An in vitro photothermal experiment was performed to measure the photothermal conversion efficiencies of both dumbbell and cylindrical silica-coated GNRs. Thermal images of their solutions (1.5 mL) were captured with an infrared thermographer (Tis65 Fluke) every 30 s under an 808 nm laser irradiation in five switchable “laser on−laser off” photothermal cycles (laser on: 300 s; laser off: cool to room temperature for 10 min). Temperature change curves were figured out from the thermal images.

An animal experiment was conducted to evaluate the photothermal potency of dumbbell silica-coated gold nanorods in vivo, and animal models of transplanted tumors on Balb/c nude mice were used in the experiment. All animal experimental procedures were approved by the Experimental Animal Center of Sun Yat-sen University and followed National Ministry of Health policies. Healthy Balb/c nude mice (4–6 weeks old, ~20 g) were kept in a pathogen-free environment during the experiments. A total of 100 μL of phosphate-buffered saline (PBS) and 4T1 murine breast cancer cells (5 × 10^6^) were injected subcutaneously into the thoracic side of Balb/c mice. After the xenografted tumor grew to approximately 60 mm^2^ for about two weeks, mice were anesthetized by intraperitoneal injection of 0.1% sodium pentobarbital (10 μL per g weight). The tumor of Balb/c mice was injected with 100 μL of dumbbell silica-coated GNRs and then irradiated with an 808 nm laser. The temperature thermal images of the tumor were recorded every 30 s with an infrared thermographer.

The 4T1 cells in cell ablation assays derived from mouse breast cancer, a metastatic tumor cell line, were inoculated on 96-well plates (8000/well) and cultured overnight at 37 °C (5% CO_2_). Dumbbell silica-coated GNRs were lyophilized and then dispersed in a phosphate buffer solution (PBS) for cell experiments. Pure PBS or dumbbell silica-coated GNRs (100 μg mL^−1^) were used to replace the original medium (1640 + 10% FBS + 1% P/S + 1% Gln), and then cultured with cells for 2 h. Then, cells were irradiated with an 808 nm near-infrared laser (0.5 W cm^−2^) for 10 min, and the medium was removed. The treated cells were washed twice with PBS and stained with calcein-AM and propidium iodide (PI) for 15 min. The cells were then fixed and imaged with a confocal laser scanning microscope (Leica SP8).

## 3. Results and Discussion

### 3.1. Machine Learning Predictions

**Figure 1:**
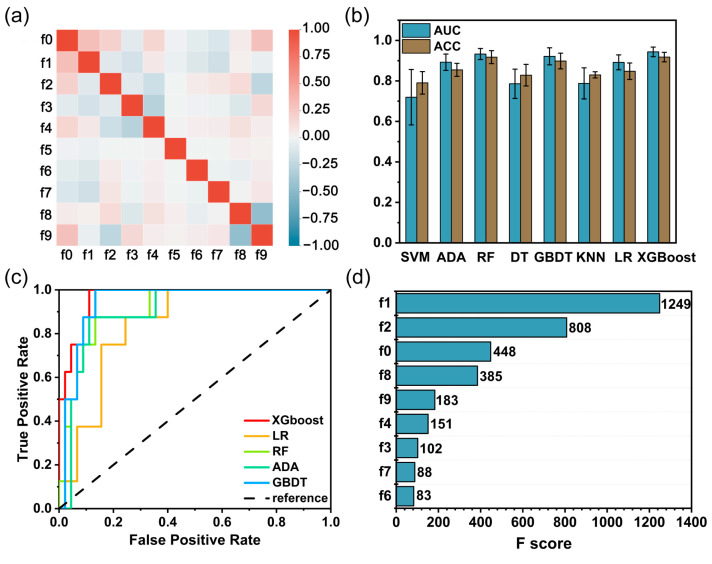
(**a**) The heat map of Pearson’s correlation coefficient matrix is among the ten features selected for the preparation of silica-coated GNRs. Feature names are listed in Table 1. (**b**) Prediction accuracy (ACC) and AUC values of various ML models. SVM: support vector machine; ADA: AdaBoost; RF: random forest; DT: decision tree; GBDT, gradient boosting decision tree; KNN, k-nearest neighbors; LR, logistic regression; and XGBoost: eXtreme gradient boosting. (**c**) ROC curves calculated with ADA, RF, GBDT, LR, and XGBoost models, respectively. (**d**) Importance scores of descriptors derived from the XGBoost model.

Well-shaped silica shells are not easily formed on the surface of GNRs uniformly, and many trial-and-error attempts are necessitated to ensure satisfactory preparation repeatability. In order to accelerate the preparation of silica-coated GNRs, ML algorithms are employed to investigate the relationship between the process and reaction outcomes of silica shells formed on GNRs. Ten features were extracted empirically from the experimental process (Table 1) and TEM images of the samples of silica-coated GNRs were pre-processed for data. These features dominate the formation of silica shells on GNRs. First, we performed a Pearson correlation analysis on these features (Figure 1a and Figure S1). The low correlation between these features suggests that there is no excessive information redundancy between them. Therefore, the independent and informative ten features that we extracted from the synthesis protocol are justified as suitable for the ML approach.

We introduced eight ML algorithms to train the data and make predictions. All parameters were initialized and tuned to find the best parameters (Table S1). The discriminative ability of the various classifiers was evaluated based on their performance on the test set. Both the AUC and ACC values of the eight classifiers are compared in Figure 1b. The results make clearly show that adaptive boosting, random forest, gradient boosting decision tree, logistic regression, and XGBoost all have both AUC and ACC values greater than 0.8. To find an accurate and easily interpretable model for the sake of application, we used the ROC curve (Figure 1c), precision, recall, and F1-score evaluation metrics (Table 2) to further evaluate the above five models. Among them, the XGBoost and random forest models achieved an accuracy of over 91%, and outstrip the other three models. Furthermore, XGBoost outperforms the random forest model on both the ROC curve and other evaluation metrics. The results above indicate that the XGBoost classifier can achieve the best performance in terms of AUC, ACC, precision, recall, and F1-score. XGBoost’s ability to handle both numerical and categorical data, its ensemble learning approach, and its regularization techniques are all factors that can contribute to its superior performance on this task. Therefore, the XGBoost classifier was selected as the best prediction model for this study.

### 3.2. Analysis and Optimization of the Preparation Process

The XGBoost model provides a built-in method to quantify the importance of features in decision-making. The processing parameter features that affect the synthesis of silica-coated GNRs are ranked in descending order of their relative importance fractions, as presented in Figure 1d. Evidently, the concentration of CTAB (f1), the volume of TEOS in a single injection (f2), and the number of GNRs (f0) are the top three dominant factors influencing the preparation of silica-coated GNRs. Among them, the concentration of CTAB is the most important factor because the surfactant CTAB is often attached to the GNRs surface as an organic template. The hydrolyzed silicon source condenses on the surface of CTAB molecules under alkaline conditions to form a silica shell. A suitable CTAB concentration facilitates the synthesis of silica-coated GNRs, while a higher CTAB concentration gives rise to the formation of silica spheres as a by-product. The amount of TEOS affects the location of silica deposition on the GNRs surface. Because the deposition of silica needs to overcome the energy barrier formed by CTAB, which is smaller at two ends of the GNRs than on their lateral sides, the silicon source is preferentially deposited at two ends of the GNRs [39]. The appropriate concentration of CTAB and TEOS, if controlled accurately, will form silica-coated GNRs with a specific dumbbell-shaped structure. The high concentration of TEOS makes condensed silica in the solution easier to overcome the barrier of CTAB formation to achieve deposition on the sides [39]. The amount of GNRs affects the thickness of the silica shell layer by influencing the amount of silicon source attached per gold nanorod particle. A higher amount of GNRs makes each gold nanorod particle absorb less silicon source and the thinner the shell formed on the gold nanorod surface and vice versa [18]. The relative importance of these processing parameters for the synthesis of silica-coated GNR materials is in accord with the intuition of experienced researchers. However, quantifying them by manual or conventional analytical methods is very challenging. XGBoost provides a simple and straightforward implementation.

Further, for other ranked features, aging time (f8), the solvent of TEOS (f9), and NaOH concentration (f4) are other factors that can predict the preparation outcomes of silica-coated GNRs (Figure 1d). From an experimental point of view, the aging time is closely related to the growth kinetics of the silica shell layer. The reaction rate of the injected shell precursor solution is fast in the initial stage (9 h) and decelerates abruptly after 12 h. The synthesized silica-coated GNRs have the same shell thickness even when stored without washing for 6 days [18]. The solvent of the TEOS solution affects the rate of hydrolysis and coalescence of TEOS. The NaOH concentration is mainly used to adjust the pH of the solution, and the rate of TEOS hydrolysis becomes larger with increasing pH, whereas the coalescence rate of TEOS is not monotonic with pH [40]. In an acidic environment, the coalescence rate of TEOS is slower [41]. The TEOS concentration (f3), reaction volume (f7), and interval time (f6) in Figure 1d also affect the synthesis of silica-coated GNRs. The TEOS concentration is the amount of TEOS mixed with the ethanol. Ethanol is used to improve the solubility of hydrophobic TEOS in deionized water. TEOS is difficult to be hydrolyzed for silica coating on GNR with just a bit of ethanol [39]. The interval between drops of TEOS solution affects the concentration of hydrolyzed TEOS in the solution. Short intervals allow hydrolyzed TEOS in solution to reach a homogeneous nucleation concentration and generate nucleation-free silica spheres. A sufficiently long interval will prevent the hydrolyzed TEOS in solution to reach the homogeneous nucleation concentration, and the hydrolyzed silicon source in solution will undergo a heterogeneous nucleation reaction, i.e., the formation of a silica layer on the surface of the GNRs [18]. As for the reaction volume, it is associated with the effect of isometric scaling of the components, which changes the interaction between the components. 

**Figure 2:**
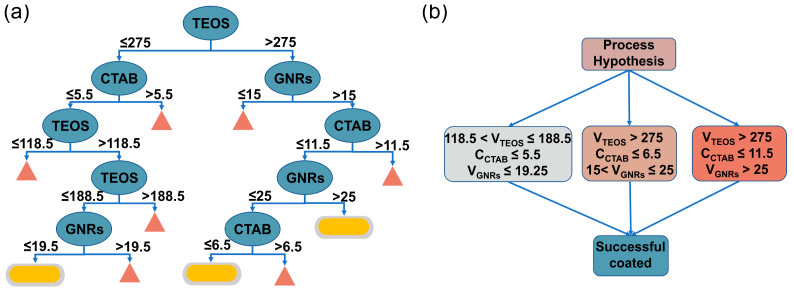
(**a**) Decision trees derived from the XGBoost model were used to classify successful and unsuccessful outcomes in silica-coated gold nanorod preparations. Ovals, triangles, and cylindricals signify decision nodes, excised subtrees, and result bins, respectively. (**b**) Graphical representation of the three process hypotheses extracted from the decision tree.

The decision rule is another characteristic that makes XGBoost highly interpretable. To gain more process insight, we derived a decision tree from the XGBoost model (Figure 2a). The decision tree shows how the decision is made to classify the reaction outcomes based on the input process parameters. The blue ovals display the decision nodes. The sketch graphs of silica-coated GNRs suggest that GNRs can be successfully coated with silica, while the orange triangles represent those that cannot prepare silica-coated GNRs or fail to decide whether they can. For the left branch of the decision tree, when the volume of TEOS in a single injection is less than 275 μL and CTAB concentration is less than 5.5 mM, the volume of TEOS in a single addition and the volume of GNRs should be adjusted to determine the preparation outcomes. For the right branch, when the volume of TEOS in a single injection is larger than 275 μL and CTAB concentration is less than 11.5 mM, as well as the volume of the GNR solution being larger than 15 mL, the outcomes are determined by sequentially adjusting the volume of GNRs and the concentration of CTAB. We can extract the process assumptions from this decision tree, including some important criteria guiding the preparation of silica-coated GNRs. As shown in Figure 2b, we can infer that the volume of TEOS in a single injection is most crucial to the final reaction outcomes. If TEOS volume in a single injection for successful synthesis of silica-coated gold is between 118.5 µL and 188.5 µL, then CTAB concentration and GNR volume in the reaction solution should preferably not exceed 5.5 mM and 19.25 mL, respectively. If TEOS volume in a single injection exceeds 275 µL, the concentration limit of CTAB can be less restrictive. For instance, when the CTAB concentration is less than 6.5 mM, then GNR volume used should be between 15 mL and 25 mL to obtain well-formed silica-coated GNRs. If the CTAB concentration is relaxed to 11.5 mM, then a GNR greater than 25 mL should be used to obtain silica-coated GNRs. 

**Figure 3:**
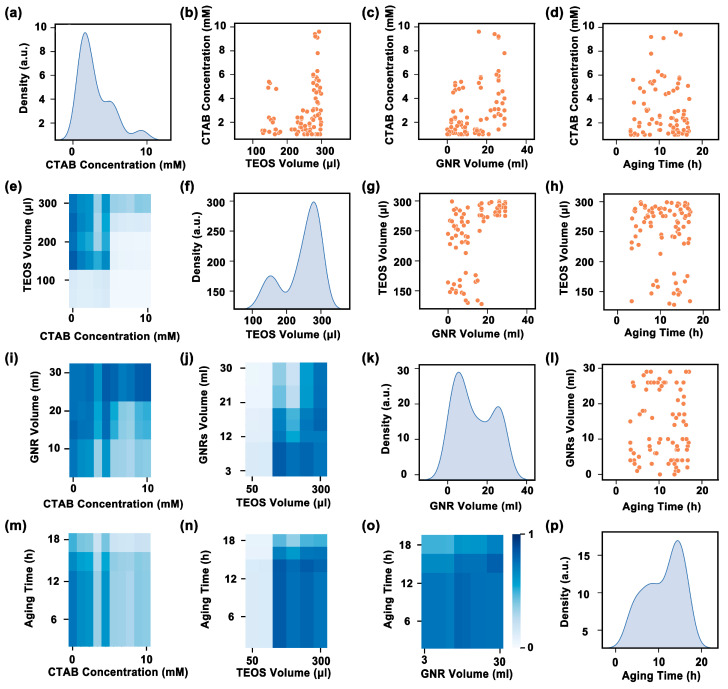
Statistical analysis based on ML predictions. Kernel density estimates of the predicted success group by the XGBoost model regarding (**a**) CTAB concentration, (**f**) TEOS volume in a single injection, (**k**) GNR volume, and (**p**) aging time, respectively. The scatter distribution of the predicting success group in the parameter space of the four foremost factors: (**b**) CTAB concentration versus TEOS volume in a single injection, (**c**) CTAB concentration versus GNR volume, (**d**) CTAB concentration versus aging time, (**g**) TEOS volume in a single injection versus GNR volume, (**h**) TEOS volume in a single injection versus aging time, and (**l**) GNR volume versus aging time. The heatmap of predictions from the trained model represented by the matrix formed by the four most important features: (**e**) TEOS volume in a single injection versus CTAB concentration, (**i**) GNR volume versus CTAB concentration, (**j**) GNR volume versus TEOS volume in a single injection, (**m**) aging time versus CTAB concentration, (**n**) aging time versus TEOS volume in a single injection, and (**o**) aging time versus GNR volume. All heatmaps share an identical color bar in values within the subgraph (**o**).

In order to obtain the optimal process parameters suggested by the XGBoost model, we randomly generated virtual experimental groups in a four-dimensional parameter space consisting of the first four significant features and input these virtual experimental groups into the model to spawn high-throughput predictions. All the predicted virtual experimental groups that could successfully synthesize silica-coated GNRs were screened out and statistically analyzed in Figure 3. Figure 3a,f,k,p are the relevant kernel function density estimation plots of four key process parameters of the successful groups. The peak positions in these plots are the most densely distributed positions of the successful groups, which can be regarded as the optimal values of the silica-coated GNR process parameters. Therefore, the optimal values of CTAB concentration, TEOS volume in a single injection, amount of GNR, and aging time are ascertained around 2 mM, 280 μL, 5 mL, and 14 h, respectively. The scatter distributions of the successful group in the parameter space consisting of pairwise features are presented in Figure 3b–d,g,h,l. They are associated with the conditions of low CTAB concentration, high TEOS amount, low GNR amount, and sufficient aging time, indicating that these conditions are in favor of the formation of silica-coated GNRs. The model can also render vivid heatmaps to visualize the probability of successful preparation in Figure 3e,i,j,m–o, where each combination of values between all pairwise features represents the probability of successful preparation of silica-coated GNRs; the success rates shown in detail in Figure S2.

### 3.3. Dumbbell Silica-Coated GNRs for Photothermal Applications

**Figure 4:**
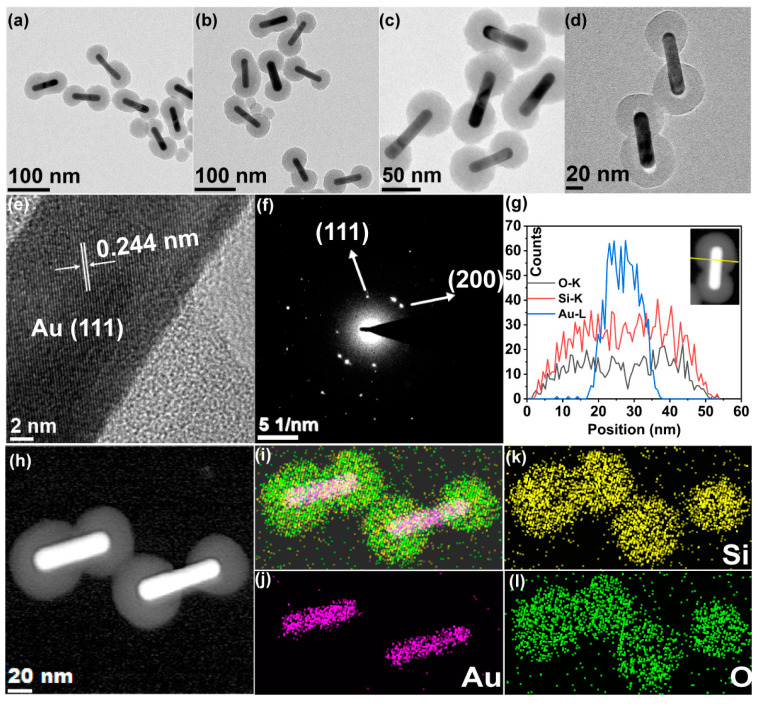
Typical TEM images of silica-coated GNRs prepared under the guidance of ML predictions. The process parameters are (**a**) 1 mM CTAB and 300 μL TEOS single injection volume, (**b**) 1 mM CTAB and 250 μL TEOS single injection volume, (**c**) 1 mM CTAB and 200 μL TEOS single injection volume, and (**d**) 1 mM CTAB and 150 μL TEOS single injection volume, respectively. (**e**) High-resolution TEM images of silica-coated GNRs. (**f**) Selected area electron diffraction pattern of silica-coated GNRs. (**g**) EDX line scanning profiles analyzed across the end spherical region as viewed along the line in the inset of the HAADF-STEM image. (**h**) STEM images before EDX mapping of the silica-coated GNRs. (**i**) The corresponding elemental mixed mapping images of elements Au (**j**), Si (**k**), and O (**l**).

High-throughput screening under the guidance of ML prediction can accelerate the preparation of silica-coated GNR materials. The resulting 35,079,400 combinations of silica-coated GNR preparations were predicted based on the input range of each feature (Table S2). Six of these experimental combinations with the highest prediction success were selected for experimental validation (Figure S3 and Table S3). Additionally, to better explain the information in the prediction combinations, four additional experimental combinations were supplemented for experimental validation in the prediction matrix consisting of the two most important features (Table S3). TEM images of the silica-coated GNRs prepared by the sol–gel method (Figure 4a–d) show that the silica has been successfully coated on GNR, indicating that machine learning-guided silica coating is feasible and effective. The silica-coated GNRs take on a dumbbell-shaped structure. HRTEM and SAED analysis (Figure 4e,f) make clear that silica-coated GNRs consist of single-crystalline gold and amorphous silica. The lattice spacing is 0.244 nm, corresponding to the (111) face of the gold crystal structure. More than one gold nanorod is present in the selected range; therefore, multiple sets of electron diffraction patterns exist. EDX line scan and elemental mapping analysis in the dark field (Figure 4g,h) offer the profiles of the distribution of O, Si, and Au elements in different regions (Figure 4i–l). 

**Figure 5:**
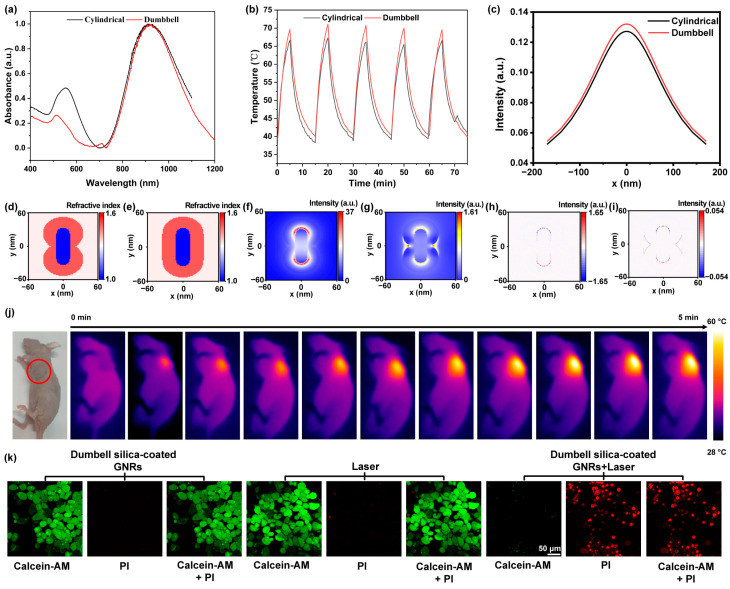
(**a**) Absorption spectra of two types of silica-coated GNRs. (**b**) Photothermal stability assay curve of dumbbell-type and cylindrical silica-coated GNRs in vitro. (**c**) Electric field enhancement around two types of silica-coated GNRs. Structure diagram of dumbbell (**d**) and cylindrical silica-coated GNRs (**e**). (**f**) Electric field distribution of dumbbell silica-coated GNRs. (**g**) Difference between the electric field of dumbbell and cylindrical silica-coated GNRs. (**h**) Charge distribution diagram of dumbbell silica-coated GNRs. (**i**) Difference between the charge of dumbbell and cylindrical silica-coated GNRs. (**j**) Photothermal images of dumbbell-type silica-coated GNRs into 4T1 breast cancer tumor mice. (Laser: 808 nm.) (**k**) Staining of the incubated 4T1 cells by calcein-AM (green) and PI (red) with different ingredients and radiation conditions. The excitation light density was set as 0.5 W cm^−2^ for in vitro cellular studies.

We employed cylindrical silica-coated GNRs as a control group to evaluate the photothermal performance of dumbbell silica-coated GNRs, both of which have longitudinal plasmon resonance absorption peaks at 907 nm (Figure 5a). A total of 1.5 mL of silica-coated GNR solution was placed in a quartz cuvette. The thermal images were captured every 30 s upon continuous irradiation with an 808 nm laser (Figure 5b and Figure S4). The results show that the dumbbell silica-coated GNRs could be heated up to 69.7 °C, which is higher than the 66.7 °C of its cylindrical counterpart after a duration of 5 min. At this point, we turned off the laser and let the solution cool off naturally for 10 min before turning it on again. After five cycles of repetition, both types of silica-coated GNRs could still reach high temperatures, indicating that they have good thermal stability. In order to theoretically interpret why dumbbell silica-coated GNRs possess an enhanced photothermal performance, we conducted an electromagnetic simulation of silica-coated GNRs with a time domain finite difference algorithm. We first calculated the electric field enhancements and charge distributions when light is horizontally incident on both silica-coated GNRs, and then figured out the difference of both charge and electric field enhancements between dumbbell and cylindrical silica-coated GNRs. Figure 5c displays the electric field enhancements along the x-axis when the incident excitation wavelength is 808 nm with a fixed y position at 158 nm and z position at 0 nm. The calculation reveals that the electric field enhancement of the dumbbell silica-coated GNR is more intensive than that of its cylindrical silica-coated counterpart. The electric field enhancements of both types of silica-coated GNRs (Figure 5d,e) are strongly distributed at two ends of the GNR with a dumbbell shape (Figure 5f and Figure S5), which is consistent with the dumbbell silica shell outside the GNR. Dumbbell-shaped silica shells also facilitate drug molecules, such as ICG accumulated at two-end shells to make the most use of local electric field enhancement. Figure 5g shows that the electric fields are significantly stronger at two ends and sides of the dumbbell silica-coated GNR than that of its cylindrical counterpart. Figure 5h,i display the charge distribution of the dumbbell-shaped silica-coated GNR and the difference in charge distribution of two kinds of silica-coated GNRs, respectively. It is well-known that an enhanced surface plasmon resonance (SPR) absorption gives rise to a larger electric field on metal nanoparticle surfaces, and contributes to the photothermal behavior of GNRs [42,43].

To evaluate the in vivo photothermal performance of silica-coated GNRs, we administrated dumbbell-shaped silica-coated GNRs into 4T1 breast tumor mice by subcutaneous injection and irradiated the tumor site with a laser beam. The temperature of the tumor site can elevate up to 60 °C within 5 min (Figure 5j and Figure S6) under an 808 nm laser irradiation, and a temperature of 60 °C is sufficient to kill the tumor cells [44,45]. Figure 5k shows calcein-AM and PI confocal fluorescence images of dead and living cancer cells. Breast cancer cells (4T1) were cocultured with a dumbbell silica-coated GNR solution for 2 h and stained after 10 min of 808 nm laser (0.5 W cm^−2^) irradiation. Dumbbell silica-coated GNRs were covalently conjugated with folic acid–polyethylene glycol (FA-PEG) molecules. Due to the high expression level of FA receptors in 4T1 cancer cells, the target FA-PEG molecules loaded on dumbbell silica-coated GNRs can promote their complex uptake by tumor cells. The dumbbell silica-coated GNRs absorbed in or on the surface of cancer cells can cause photothermal damage to cancer cells when they are exposed to an 808 nm laser. Live cells labeled with calcein-AM showed green color, while dead cells labeled with PI showed red fluorescence. Cell imaging revealed that cancer cells, under either laser irradiation alone or simply dumbbell silica-coated GNRs without laser irradiation, could survive in the medium. Almost all cancer cells were killed by the combination of laser irradiation with dumbbell silica-coated GNRs. These results clearly demonstrate that the photothermal impact of dumbbell silica-coated GNRs can kill cancer cells even at a low excitation power. In brief, dumbbell silica-coated GNRs exhibit superior photothermal performance and a good prospect for tumor ablation therapeutics. Systematic preclinical investigations on dumbbell silica-coated GNRs are necessarily carried out further.

## 4. Conclusions

We report the preparation of well-shaped silica-coated gold nanorods (GNRs) assisted by machine learning (ML) prediction and identify a unique dumbbell structure with excellent optical properties and promising photothermal potential for applications. A total of 306 sets of silica-coated GNRs prepared under different conditions were characterized and adopted as training datasets for ML modeling with eight algorithms. Among them, the XGBoost algorithm outstrips others with the best prediction accuracy of over 0.91. According to ML-derived feature importance and decision trees, the current study proffers a comprehensive interpretation of the process conditions for fabricating silica-coated GNRs. Cetyltrimethylammonium bromide (CTAB), tetraethyl orthosilicate (TEOS) concentrations, and GNR amounts are the three most important features that affect the final preparation of silica-coated GNRs. The decision trees derived from XGBoost were employed to optimize the preparation process of silica-coated GNRs. Using high-throughput predictions, a statistical analysis of the virtual success group was attempted to determine the optimal conditions, and then the unique dumbbell-shaped silica-coated GNRs were successfully prepared under the guidance of the XGBoost model. In vitro and in vivo experimental tests indicate that the photothermal performance of dumbbell-shaped silica-coated GNRs are superior to that of the conventional cylindrical silica-coated GNRs, which was also proved via the time domain finite difference simulation. Pursuant to the simulation, the local electric field enhancement of dumbbell silica-coated GNRs is more intensive than that of its cylindrical counterpart, leading to enhanced surface plasmon resonance absorption on GNRs with a stronger photothermal effect for a better tumor-ablating therapy application. The method reported in this study can also be extended to design and develop other composite nanostructure materials.

## Figures and Tables

**Table 1:** The description of feature names.

Code Name	Process Parameters
f0	The number of gold nanorods
f1	The concentration of cetyltrimethylammonium bromide (CTAB) in the solution
f2	The volume of tetraethyl orthosilicate (TEOS) in a single injection
f3	The concentration of the TEOS solution
f4	The solvent of the TEOS solution
f5	The concentration of the sodium hydroxide (NaOH) solution
f6	The rate of stirring mixtures in the solution
f7	The interval between drops of the TEOS solution
f8	The total volume of the solution
f9	The age of the solution

**Table 2:** Evaluation metrics of five ML models.

	AUC	ACC	Precision	Recall	F1 Score
ADA	0.892706	0.854736	0.59784	0.752517	0.665029
RF	0.933072	0.917686	0.774662	0.752517	0.763268
GBDT	0.921759	0.898529	0.727928	0.772071	0.748218
LR	0.89211	0.848096	0.593264	0.518264	0.551995
XGboost	0.943967	0.917935	0.812165	0.768881	0.786131

## Data Availability

The data that support the findings of this study are available within the article and its Supplementary Material. Further data are available from the corresponding author upon reasonable request.

## Supplementary Materials

The following supporting information can be downloaded at https://www.mdpi.com/article/10.3390/nano13061024/s1, Figure S1: The heat map of Pearson’s correlation coefficient matrix among the features selected for the preparation of silica-coated GNRs; Figure S2: The heatmap of predictions from the trained model represented by the matrix formed by the four most important features: (a) TEOS volume in a single injection versus CTAB concentration, (b) GNR volume versus TEOS volume in a single injection, (c) aging time versus GNR volume, (d) GNR volume versus CTAB concentration, (e) aging time versus CTAB concentration, and (f) aging time versus TEOS volume in a single injection; Figure S3: TEM images of the validation experiments. (a–f) are the TEM images of the experimental groups corresponding to the IDs in Table S3, respectively; Figure S4: The temperature rising curve of photothermal images of dumbbell silica-coated GNRs; Figure S5: Electric field enhancement and charge distribution in cylindrical silica-coated GNRs; Figure S6: In vivo photothermal temperature rising curve in the tumor site of mice; Table S1: Optimal hyperparameters obtained through 5-fold GridSearchCV procedures; Table S2: Parameter space for each experimental variable; Table S3: Parameters of the validation experiments obtained with high throughput prediction and combined effects.
