# Supplementary material for: Machine Learning Predicting Optimal Preparation of Silica-Coated Gold Nanorods for Photothermal Tumor Ablation
Source: Nanomaterials (Basel). 2023 Mar 12;13(6):1024. doi: 10.3390/nano13061024 (PMC10059579; doi:10.3390/nano13061024)
Supplement: Supplementary file 1 [file nanomaterials-13-01024-s001.zip › nanomaterials-2232531-supplementary.pdf]

nanomaterials

Supplementary Materials

Machine Learning Predicting Optimal Preparation of Silica-Coated Gold Nanorods for Photothermal Tumor Ablation

Jintao Zhang [1], Jinchang Yin [1,2,*], Ruiran Lai [1], Yue Wang [1], Baorui Mao [1], Haonan Wu [1], Li Tian [3] and Yuanzhi Shao [1,4,*]

1 State Key Laboratory of Optoelectronic Materials and Technologies, School of Physics, Sun Yat-sen University, Guangzhou 510275, China
2 Institute of Biological and Medical Engineering, Guangdong Academy of Sciences, Guangzhou 510316, China
3 State Key Laboratory of Oncology in South China, Collaborative Innovation Center for Cancer Medicine, Sun Yat-sen University Cancer Center, Guangzhou 510060, China
4 Guangdong Provincial Key Laboratory of Magnetoelectric Physics and Devices, Sun Yat-sen University, Guangzhou 510275, China
* Correspondence: yinjch6@mail.sysu.edu.cn (J.Y.); stssyz@mail.sysu.edu.cn (Y.S.)

Table S1. Optimal hyperparameters obtained through 5-fold GridSearchCV procedures.

Models	Full Name	Hyperparameters
KNN	k-Nearest Neighbors	n_neighbors = 10
LR	Logistic Regression	C = 10, max_iter = 100
SVM	Support Vector Machines	C = 10, gamma = 1, kernel = 'rbf'
DT	Decision Tree	max_depth = 9
RF	Random Forest	n_estimators = 400, max_depth = 8
ADA	AdaBoost	DecisionTreeClassifier(max_dep th = 6), learning_rate = 0.1, n_estimators = 450
GBDT	Gradient Boosting Decision Tree	learning_rate = 0.1, max_depth = 3, n_estimators = 150
XGBoost	eXtreme Gradient Boosting	max_depth = 5, n_estimators = 419, min_child_weight = 1, subsample = 0.9, colsample_bytree = 0.9, learning_rate = 0.01

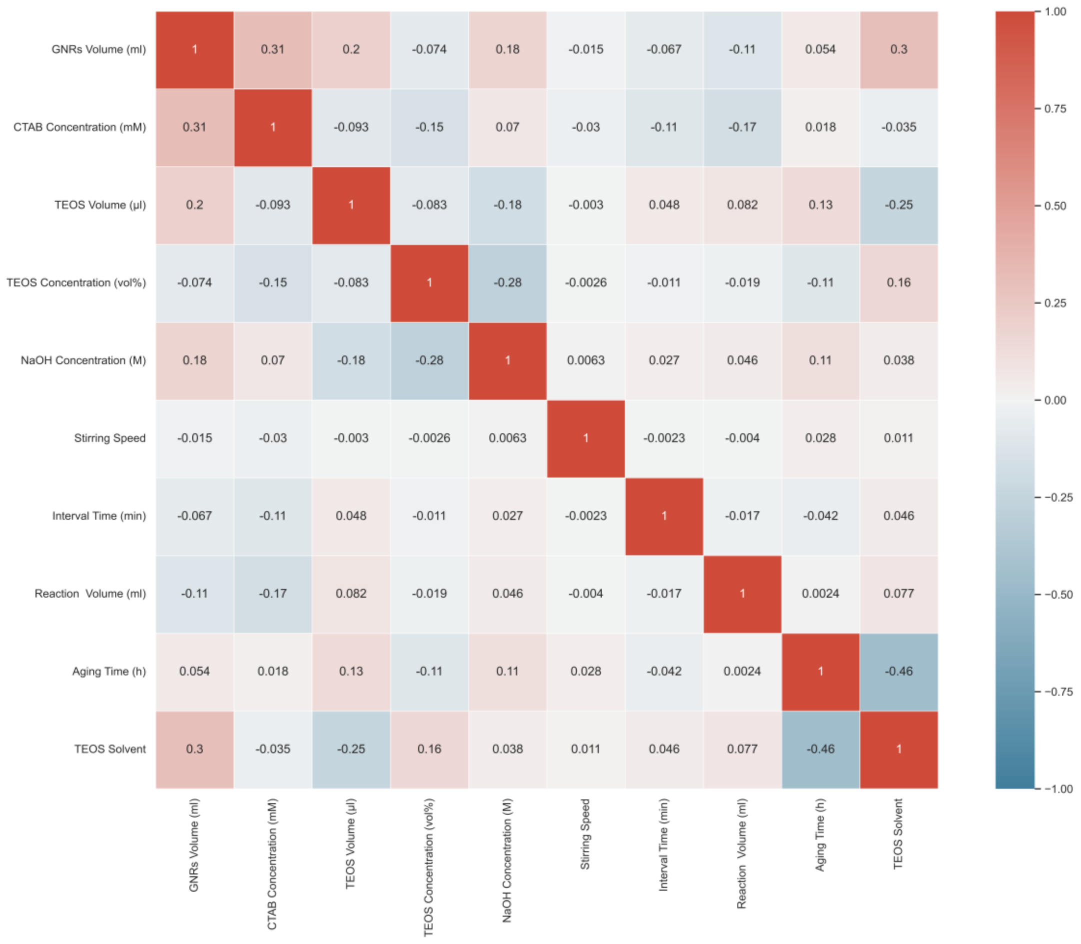

Figure S1. The heat map with values of the Pearson's correlation coefficient matrix among the features selected for the preparation of silica-coated GNRs.

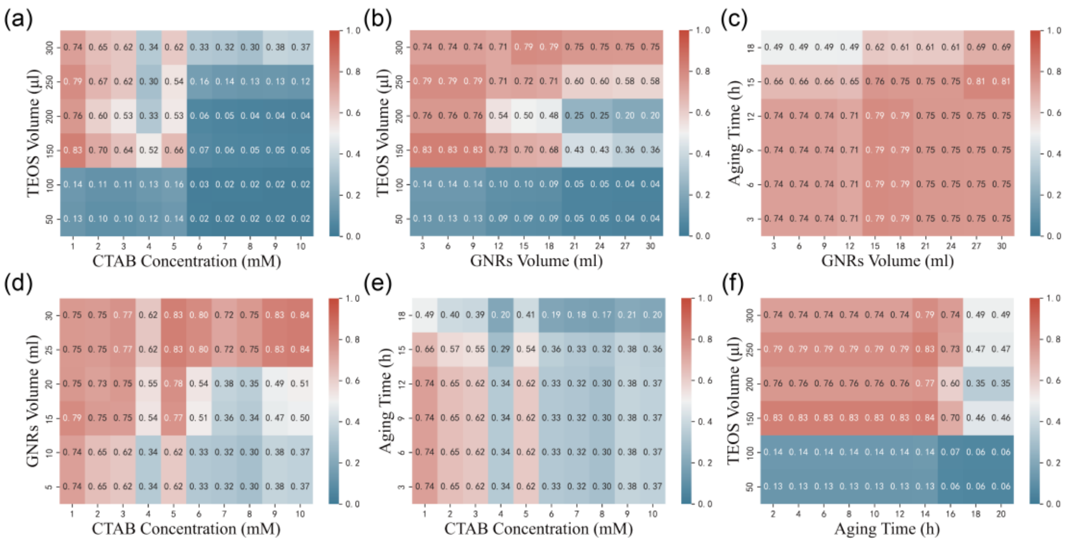

Figure S2. The heatmap of predictions from the trained model represented by the matrix formed by the four most important features: (a) TEOS volume in a single injection versus CTAB concentration, (b) GNRs volume versus TEOS volume in a single injection, (c) Aging time versus GNRs volume, (d) GNRs volume versus CTAB concentration, (e) Aging time versus CTAB concentration, (f) Aging time versus TEOS volume in a single injection.

Table S2. Parameter space for each experimental variable.

Process parameters	Prediction space
The amount of gold nanorods	[1,2,3, 4,5,6,7,8,9,10,11,12,13,14,15,16,17,18,19,20,21,22,23,24,25,26,27,28,29,30]
The concentration of cetyltrimethylammonium bromide (CTAB) in the solution	[0,1,2, 3, 4,5,6,7,8,9,10,11,12,13,14,15,16,17,18,19]
The volume of tetraethyl orthosilicate (TEOS) in a single injection	[50,100,150, 200,250,300,350,400,**450**,50**0**]
The concentration of TEOS solution	[0.25,0.5,0.75]
The concentration of Sodium hydroxide (NaOH) solution	**[**0.025,0.05,0.1]
The rate of stirring mixtures in the solution	[0,1,2]
The Interval between drops of TEOS solution	[20,30,40,50]
The total volume of the solution	[15,20,30,40]
The age of the solution	[6,9,12,14,15,16,18]
The solvent of TEOS solution	[0.2818, 0.08888889][a]

[a]0.2818 and 0.08888889 in the solvent represent the values after target coding for the two solvents ethanol and isopropanol for TEOS, respectively.

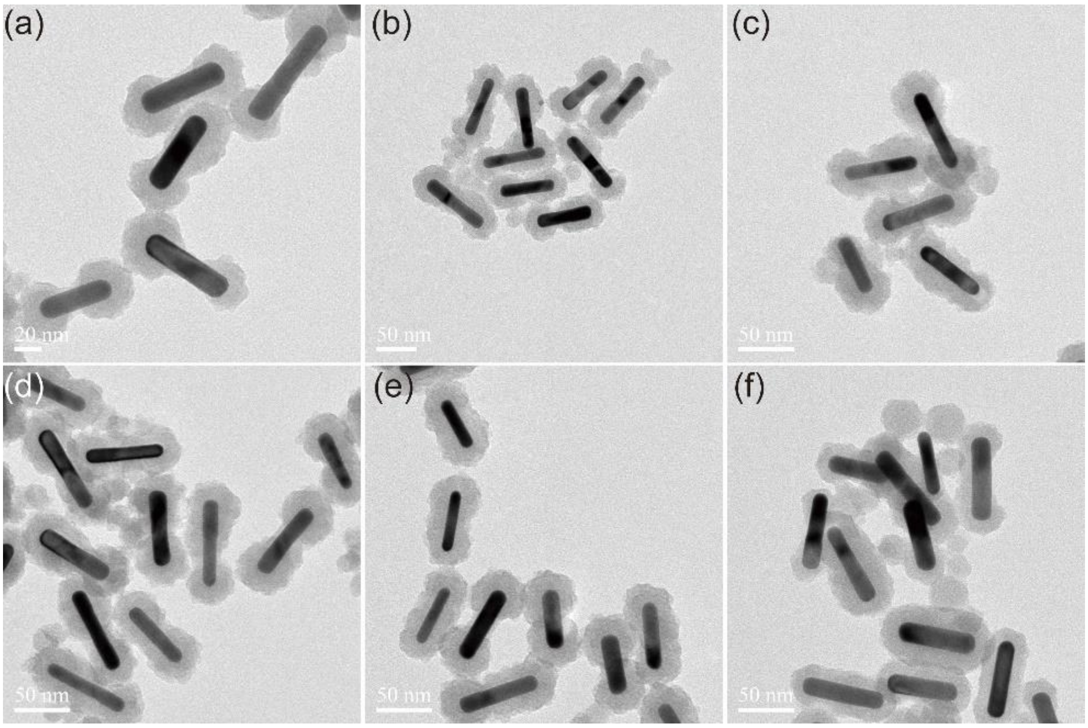

Figure S3. TEM images of the validation experiments. (a), (b), (c), (d), (e) and (f) are the TEM images of the experimental groups corresponding to the IDs in Table S3, respectively.

Table S3. Parameters of the validation experiments obtained with high throughput prediction and combined effects.

ID	V_{GNRs}	C_{CTAB}	V_{TEOS}	C_{NaOH}	$N_{Stirring}$	$t_{Interval}$	t_{Aging}	Probability
A	29	5	300	0.1	1	50	16	0.92
B	29	5	400	0.1	2	50	16	0.92
C	28	5	450	0.1	2	50	16	0.92
D	28	5	500	0.1	1	50	16	0.92
E	27	5	450	0.1	1	50	16	0.92
F	26	5	450	0.1	0	50	16	0.92
G	10	1	150	0.1	1	30	12	0.83
H	10	1	200	0.1	1	30	12	0.76
I	10	1	250	0.1	1	30	12	0.79
J	10	1	300	0.1	1	30	12	0.74

Other parameters not listed in the table are identical, e.g. TEOS concentration is 25 vol%, reaction volume is 15 mL, and the solvent for TEOS is alcohol.

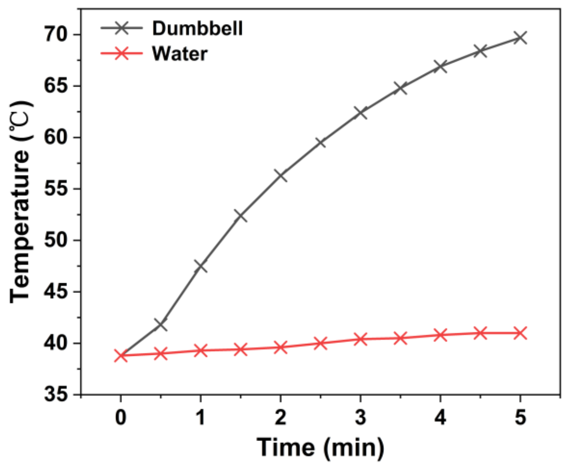

Figure S4. The temperature-rising curve of photothermal images of dumbbell silica-coated GNRs.

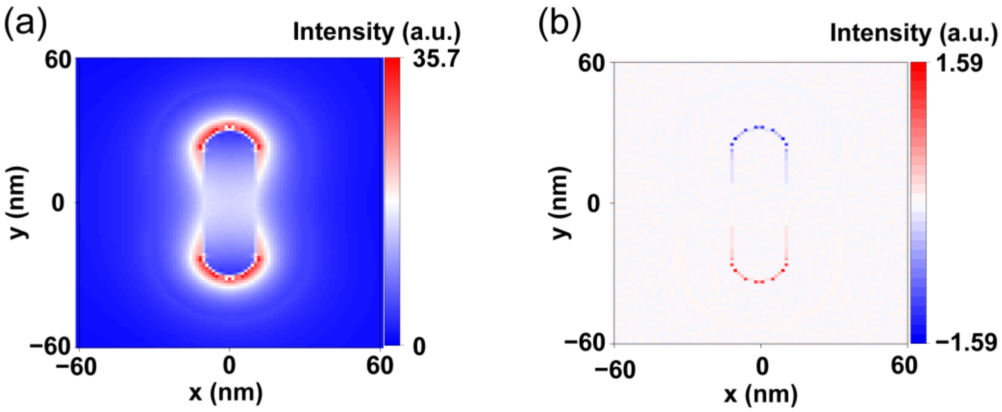

Figure S5. Electric field enhancement and charge distribution in cylindrical silica-coated GNRs.

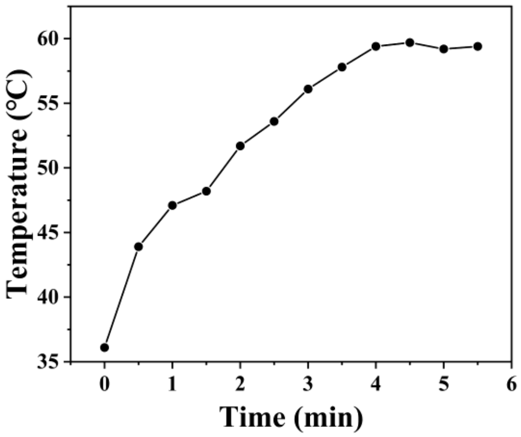

Figure S6. *In vivo* photothermal temperature rising curve in tumor site of mice.
